# Professors Prioritize Increasing Female Retention in Academic Physics Over Advisee’s Interests

**DOI:** 10.3389/fsoc.2021.751703

**Published:** 2022-02-02

**Authors:** Kimberlyn Bailey, David Horacek, Steven Worthington, Melissa Schmitz

**Affiliations:** ^1^ Department of Biostatistics, Harvard TH Chan School of Public Health, Boston, MA, United States; ^2^ C2 Education, Bethesda, MD, United States; ^3^ Institute for Quantitative Social Science, Harvard University, Cambridge, MA, United States; ^4^ Department of Physics, Le Moyne College, Syracuse, NY, United States

**Keywords:** gender bias, women in STEM, underrepresentation of women, physics, diversity, stem

## Abstract

Decades of initiatives have striven to fix the so-called “leaking pipeline” problem—persistent high attrition of women from the career/educational path toward STEM professorship. Though these initiatives call on academics to increase female retention along this path, it remains unknown whether academics actually prioritize this goal. To investigate this, we tested whether academics would prioritize female retention at the cost of a competing goal when giving career advice to students at risk of leaving the “pipeline.” We present results from a national survey in which United States professors (n = 364) responded to vignettes of three hypothetical undergraduates, rating the extent to which they would encourage or discourage each student from pursuing a PhD in physics. Professors were randomly assigned vignettes with either male or female gender pronouns. Two vignettes featured students who cogently explained why remaining in the physics pipeline would not match their individual goals and interests, while another vignette presented a student with goals and interests that clearly matched pursuing physics graduate school. Professors who received female-gendered vignettes were thus forced to choose between prioritizing striving to increase female retention in physics and acting in the best interest of the individual student. We present evidence that professors seem prepared to encourage women more strongly than men to remain in physics, even when remaining is contrary to the stated goals and interests of the student: Our logistic regression results suggest that professors have higher odds of encouraging women over men, net of vignette and other controls. We also find that male professors have higher odds of encouraging undergraduates and find no evidence that, relative to non-STEM professors, STEM professors have higher odds of encouraging women over men.

## 1 Introduction

The metaphors we choose to describe an issue both shapes and reveals how we think about it (Morgan, 1998). In United States national reports, popular news and scholarly debate on female underrepresentation in science, technology, engineering and mathematics (STEM), the ubiquitous metaphor is the leaky pipeline (Alper, 1993; Blickenstaff, 2005; Ceci et al., 2014; NRC, 2006; IM2, 2007; Harmon, 2018; Williams and Massinger, 2016).

The metaphor captures troubling, persistent patterns of female attrition on the path toward tenured STEM professorship in the United States, with attrition conceptualized as “leaks” from the pipeline. In some fields in the United States, like physics, women have long been underrepresented at each career/educational step. Women comprise only 16% of physics faculty and leave at much higher rates than men at early stages of physics education (AIP, 2019), with only 20% of bachelors degrees in physics awarded to women (Society, 2019). Even in other STEM fields in the United States, like biology, that have made considerable progress toward gender balance, underrepresentation persists at the senior-most career milestones (Ceci et al., 2014).

A wealth of research conducted in the Western world suggests this longstanding underrepresention is due to a thicket of entrenched injustices hindering women in STEM, causing the so-called “leaks.” Stereotypes that link men but not women with scientific ability (Bennett, 1996; Bennett et al., 2007; Tiedemann, 2000), social climates unwelcoming to women (Bilimoria et al., 2008; Settles et al., 2006) and gender bias in hiring (Moss-Racusin et al., 2012; Reuben et al., 2014) are only a few such hindrances. Widespread public enthusiasm and numerous institutional initiatives have arisen in the United States to fight these injustices, especially in academia, where many scholarships, policies and workshops to support women in STEM are now common.

Although efforts to support women in STEM seek to redress the injustices responsible for underrepresentation, they often measure their success in terms of representation itself. United States universities, agencies, popular news and scholars closely track changes in female representation, celebrating gains (Hill et al., 2010; NRC, 2010; NSF, 2010; Smyth and Nosek, 2015; Kang and Banaji, 2006; Turner et al., 2008; Niederle et al., 2013; EOP, 2012; Harmon, 2018; Williams and Massinger, 2016; NAS, 2020). Scholars and reports from United States agencies and organizations highlight stagnating increases in female retention, which they cite as reasons to redouble support (AIPAAU, 2015; Kahn and Ginther, 2018; Kahn and Ginther, 2019). In this way, efforts to rectify gender injustices in STEM have created a strong imperative for everyone, and especially academics, to try to increase female retention. Given this focus on retention, the popularity of the leaky pipeline metaphor is unsurprising–the metaphor implores us to “seal the leaks.”

Echoing the push to increase female representation in academic STEM, do academics prioritize “sealing the leaks?” If something is a priority, we place more importance on it than other considerations, trading off those other considerations for the sake of the thing we prioritize. “Work-life balance,” for example, exemplifies this idea. What makes both “life” and “work” priorities is that we place enough importance on each of them to constantly trade-off one at a cost to the other. A clear indication that academics prioritize female retention in STEM academia would be if, when advising students on career choices, academics strive to increase female retention at a cost to a competing goal they also consider important.

When we advise someone on career/educational choices, we often prioritize that individual’s best interests. It is popularly believed that the careers to which we “match” are those in our best interests to pursue (Zichy and Bidou, 2007; Christen and Bolles, 2011; Tieger et al., 2014). When seeking to “match,” we fine-tune our career/educational advice with questions like “Do your interests match career X?” and “Does career X match your personal goals?” If the answer to either of these questions is no, many would move to recommend taking option X off the table for the best interests of the individual. We refer to this priority–striving to “match” individuals to career/educational paths out of the best interest of the individual–as the *matching mindset*. A strong motivation to seal the “leaks,” by contrast, could propel us to keep “mismatch” career options on the table if the individual in question is a woman reconsidering plans in STEM. We refer to this goal–striving to increase female representation on the path toward tenured STEM professorship–as the *female retention mindset*.

To assess whether academics prioritize female retention, we tested whether academics would act on the female retention mindset at a cost to a competing goal–in this case, the matching mindset. The key difference between these two mindsets is this: While the matching mindset implores academics to encourage pursuing STEM academia based on to the degree to which someone–be it a male or female–seems to “match” with academic STEM, the female retention mindset implores academics to change their advice for women. In effect, a trade-off between these two mindsets would manifest as follows: Preferentially encouraging women to pursue academic STEM more than otherwise identical men across varying degrees of “match”/“mismatch” with academic STEM.

To test if academics would make such a trade-off, we used a national survey of professors (n = 364) from United States colleges and universities (Bailey et al., 2019). We presented professors vignettes of undergraduates reconsidering plans to go to STEM graduate school and asked them to rate the extent to which they would encourage or discourage the undergraduates to follow through with their plans.

To detect the female retention mindset, professors were randomly assigned male or female student vignettes. Our female vignettes depict undergraduates who are well-known as crucial “leaks”: undergraduate women considering physics graduate school. The jump from college to graduate school is an important juncture on the path toward STEM professorship within the United States. This juncture marks the start of training devoted expressly to becoming an academic. For STEM fields with persistent female underrepresentation at the undergraduate level, like physics, this juncture accounts for a steep decline in female representation (Ceci et al., 2014). Physics is also a field that receives considerable resources for the purpose of increasing female representation. We, in short, presented professors with women for which the imperative to “seal the leaks” is likely strong to test whether professors would act on it.

To force a trade-off between the matching mindset and the female retention mindset, we presented each professor with three vignettes of undergraduates reconsidering physics graduate school. One vignette depicts a student who shows signs that he/she is well-“matched” to a career in academic physics. He/she has plentiful enthusiasm and merit, but, harboring unjustified self-doubt, is in need of a bit of encouragement to go to physics graduate school. The other two vignettes describe students who show signs physics graduate school is not in his/her best interests. One undergraduate presents strong signs that he/she has permanently lost interest in physics, and the other presents a reason pursuing a physics PhD conflicts with his/her personal goals. Dead-set on becoming a physics professor, but only accepted to PhD programs unlikely to lead to professorship, he/she does not want to enroll.

Within the efforts to support women in STEM, female underrepresentation at each step on the path toward tenured STEM professorship is treated as a key metric of social progress. We reasoned that, in line with this focus on retention, female retention is a priority in the minds of academics. One clear sign academics hold such a priority would be if academics act on the female retention mindset, shaping how they advise students on career/educational choices, at a cost to a second priority that also shapes their advice. To test this, we designed our vignettes to force professors to choose between these two mindsets. We depicted two undergraduates who shows strong signs of a “mismatch” to academic physics and one undergraduate who shows strong signs of a “match,” expecting to spur professors’ advice toward opposite ends of the encourage/discourage spectrum and randomly assigned professors male or female vignettes. We expected that professors will have higher odds of encouraging a female undergraduate on the fence about physics graduate school than males, even accounting for whether the undergraduate in question is a “match” or not. We present this as our first hypothesis:

**H1:** Professors have higher odds of encouraging female undergraduates than male undergraduates, net of controls.

Studies have shown that attitudes about women in STEM differ between men and women in the United States Some work has suggested male and female STEM faculty differ as to whether they give preference to males or females in hiring decisions (Sheltzer and Smith, 2014; Williams and Ceci, 2015). Other work has shown the gender of university administrators is predictive of their preferred strategy to increase female STEM representation (Williams et al., 2017). We thus expected to find that professor gender impacts a professor’s choice to encourage or discourage, stated here as our second hypothesis:

**H2:** The odds of a professor encouraging a given undergraduate differs between male and female professors, net of controls.

Though there is widespread enthusiasm in United States academia to support women in STEM, the STEM academy is especially rich with zeal and resources devoted to that mission. By virtue of that concentrated enthusiasm, we reasoned that the career/educational advice of STEM professors would be more closely aligned to the female retention mindset than the matching mindset, relative to their non-STEM colleagues. We thus expected to find that a professor’s affiliation or lack thereof with a STEM department would influence his or her decision to encourage. We also expected to find that, compared to non-STEM professors, STEM professors would encourage female students more often than male students, even when accounting for whether the undergraduate in question is a good “match” for academic physics or not and other predictors. We present this as our third and fourth hypotheses:

**H3:** Professors in STEM department(s), more than those not in STEM-department(s), have higher odds of encouraging undergraduates, net of controls.
**H4:** Professors in STEM department(s), more than those not in STEM-department(s), have higher odds of encouraging female undergraduates than male undergraduates, net of controls.

## 2 Methods and Materials

### 2.1 Survey

Our contact list of professors was collected from directories on university websites from a total of 120 universities during spring 2018. To ensure our professors hailed from geographically diverse set of universities with varying levels of institutional prestige, universities were selected from United States News’ 2018 lists for top public, national, liberal arts and regional universities, with 20 universities randomly chosen from each list. For this randomization, we randomly sampled without replacement from 1 to the length of the list using the sample function in R (R Core Team, 2021). Insofar as our vignettes depicted undergraduates, we surveyed professors only from departments that typically teach and work with undergraduates. (Departments of medicine and law, for example, were not used.) A total of 12,987 professors were contacted via email. Because the topic of our study–female representation in academia–could plausibly provoke social desirability bias, we veiled the precise purpose of our study, describing it as an investigation of “social influences on educational choices”. We collected a total of 429 responses with a 3.3% response rate. Professors who did not complete the entire survey were excluded from the final data set, producing a final count of 364 professors. Our survey was approved by the Institutional Review Board for the Protection of Human Subjects of Le Moyne College in Syracuse, NY. We created our survey with Survey Monkey.

**FIGURE 1:**
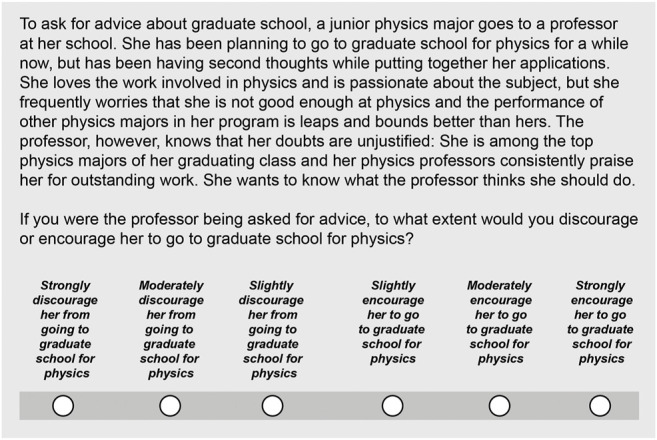
Example of a vignette and question pair as presented to respondents in our survey. This is the “match” vignette for a hypothetical female undergraduate. Each professor was presented vignettes of an additional two hypothetical undergraduates (“Conflicting personal goals” and “Loss of interest” vignettes). The gender of the undergraduate was randomly assigned, and all three vignettes shared the same gender.

Respondents were presented with three vignettes, each describing the situation of a hypothetical student asking for advice. With each vignette, respondents were prompted with the question: “If you were the professor being asked for advice, to what extent would you discourage or encourage him/her to go to graduate school for physics?” (“him” and “her” set to match the gender of the vignette). To respond, they were provided a six point scale, with three possible magnitudes of each encouragement and discouragement: slightly, moderately, strongly. For each respondent, the vignette order was randomized. Respondents were randomly assigned male and female versions of the vignettes, differentiated only by male and female pronouns. In both cases, randomization was achieved using the built-in survey features in Survey Monkey. The gender of the vignettes was the same across all three vignettes (i.e. Each respondent was either assigned all female or all male vignettes). An example of this question and vignette pair in presented in Figure 1. Additionally, respondents were asked for their gender and to list the department(s) with which they are affiliated at their universities. Departmental affiliations were coded as either STEM or non-STEM, with those professors who had at least one departmental affiliation from a STEM department classified as STEM professors. In total, 113 professors worked in STEM and 251 worked in non-STEM departments; 171 were male and 193 were female.

### 2.2 Vignettes

Though vignettes are an imperfect tool to investigate what people believe and how they would act, our research objectives made vignettes an apt choice. We recognize that advisory conversations between professors and students are more nuanced in real life than what was captured in our survey. A professor would likely engage the student in conversation, settling on what they would advise the student only after considering many details. A professor’s advice would likely be nuanced in a way that a one-dimensional scale could not capture. Ultimately, however, our objective was not to get an accurate picture of how professors act in these advisory roles. Instead, we wanted to get a snapshot of beliefs that would likely guide conversations of these sort–beliefs that would likely influence a course-grained judgment to encourage or discourage the undergraduate to remain on the STEM academic path. Indeed, vignettes are often used for this reason (Hughes, 1998)—to “clarify the judgment principles employed” in a given situation, rather than to “mirror the real world” (Rossi and Alves, 1979, p.954).

Key to our study was assessing the impact of student gender on professors’ advice while accounting for how their advice was affected by whether a student “matched” to a career in academic physics or not (See introduction). Accordingly, a key dimension by which we wanted our hypothetical students to vary considerably was the degree to which each student was a good “match” for a career in academic physics. Constructing a case that most would consider a good “match” was easy to achieve with a single vignette depicting a student both exceptionally good at and enthusiastic about physics (“Imposter syndrome” vignette). What constitutes a bad “match” for a given career–and for academic physics in particular–is, however, more subjective. For this reason, we included not one, but two vignettes we judged to be cases of students who, by popular ideas of what constitutes a good “match,” showed clear signs of a “mismatch” (“Loss of interest” and “Conflicting personal goals” vignettes). We reasoned that, between those two vignettes, our survey would be able to capture some of the effect of a “mismatch” on professors’ choices.

We crafted our vignettes to strike a balance between two ends. First, the vignettes are meant to have enough nuance to portray students who seem realistic, so that professors could respond to them as they would to real students. Second, the vignettes needed to give readers strong reasons to interpret each student as the “match” or “mismatch” that we sought to depict. Our vignettes are shown below for the male version of the vignettes (i.e., with male pronouns). We title each vignette as the reason the given student was reconsidering physics graduate school.

#### 2.2.1 Imposter Syndrome

To ask for advice about graduate school, a junior physics major goes to a professor at his school. He has been planning to go to graduate school for physics for a while now, but has been having second thoughts while putting together his applications. He loves the work involved in physics and is passionate about the subject, but he frequently worries that he is not good enough at physics and the performance of other physics majors in his program is leaps and bounds better than his. The professor, however, knows that his doubts are unjustified: He is among the top physics majors of his graduating class and his physics professors consistently praise him for outstanding work. He wants to know what the professor thinks he should do.

#### 2.2.2 Conflicting Personal Goals

A senior physics major needs to make a decision about graduate school and goes to a professor at his school to ask for advice. He tells his professor that he is passionate about physics and has, for years, envisioned a career as a physics professor for himself. Unfortunately, his application was not strong enough to get him accepted into a highly ranked Ph.D. program. The one university that accepted him rarely produces graduates that go on to become professors. He expects graduate school will be a hard venture and doesn’t think the struggle would be worth it if he does not go on to become a physics professor. He wants to know what the professor thinks he should do.

#### 2.2.3 Loss of Interest

A junior physics major is having doubts about his plans to go to graduate school for physics and goes to a professor at his school to ask for advice. He is one of the top students in his class, professors consistently praise his work, and he is confident that he could handle graduate school in physics. He is concerned, however, that his enthusiasm for physics has dwindled, and that, for the past few semesters, he feels like he is just going through the motions and not enjoying his classes. He can’t picture how graduate school would revive his excitement for physics. Initially, physics used to be as much as a hobby as it was his major, but now he does not touch the subject outside of his assigned coursework. He wants to know what the professor thinks he should do.

### 2.3 Statistical Analyses

To test our three hypotheses, we used mixed effects logistic regressions with professor’s choice to encourage an undergraduate as the outcome, vignette type, undergraduate gender and professor gender as predictors, and random intercepts grouped by professor to account for each professor making multiple choices. For ease of interpretation, we exponentiated raw regression coefficients to produce adjusted odds-ratios for which we report point and interval (95% confidence) estimates. The statistical significance of individual coefficients was determined via two-tailed likelihood ratio tests (LRT) and overall model goodness-of-fit via LRTs of the target model compared with a null, intercept-only, model. All statistical analyses were performed in R v. 4.1 (R Core Team, 2021).

## 3 Results

In this study, we aimed to assess whether academics prioritize increasing female retention on the path toward STEM professorship. As one way to detect such a priority, we sought to test if academics would strive to increase female retention at the cost of a second goal that they consider important–in this case, striving to “match” individuals to careers. Our approach, however, presumes that the professors’ advice was substantially affected by whether an undergraduate showed signs of “matching” or “mismatching.” In other words, that professors’ advice shifted toward opposite ends of the encourage/discourage spectrum for “match” and “mismatch” vignettes. Our results suggest that our study design achieved that end. Vignette type substantially affected professors’ choices. Professors almost unilaterally encouraged the “match” undergraduate (“Imposter syndrome” vignette: 98% encouragement) while their reactions were mixed to the “mismatch” undergraduates (“Loss of interest” vignette: 38% encouragement, “Conflicting personal goals” vignette: 53% encouragement).

**FIGURE 2:**
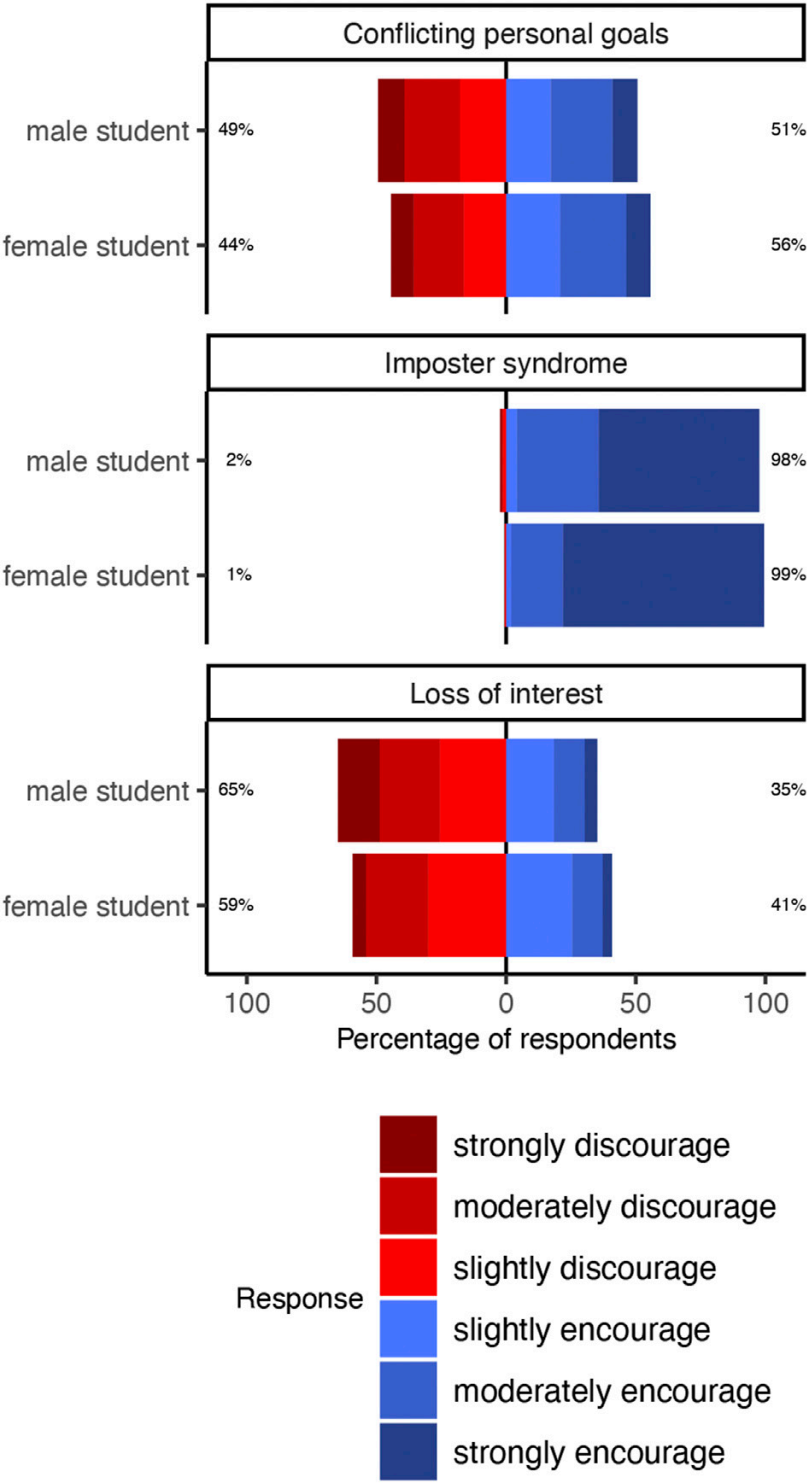
Effect of undergraduate gender and vignette on professors’ ratings of the extent to which they would encourage or discourage each undergraduate to follow through with plans to go to physics graduate school. Vignettes are titled as the reason each undergraduate presented for reconsidering physics graduate school. Percentages indicate the proportion of professors who encouraged (blue) or discouraged (red) in response to each possible pairing of undergraduate gender and vignette. A substantially greater proportion of professors chose to encourage undergraduates we designed to present strong signs that going to physics graduate school was in the student’s best personal interests (“Imposter syndrome” vignette) relative to vignettes we designed to depict the opposite (“Loss of interest” vignette and “Conflicting personal goals” vignette). Across all vignettes, a larger proportion of professors chose to encourage women undergraduates than men.

**FIGURE 3:**
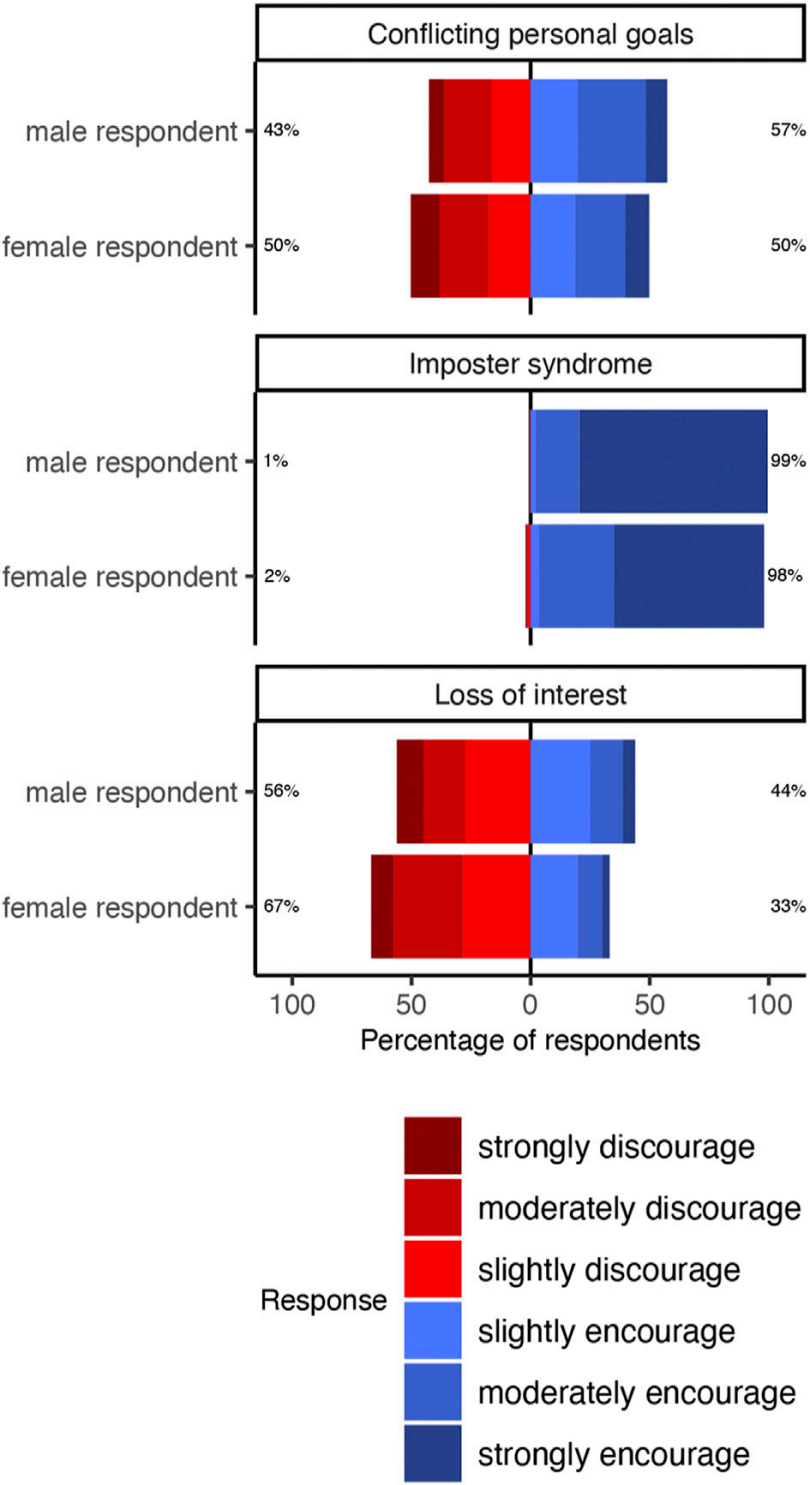
Effect of professor gender and vignette on professors’ ratings of the extent to which they would encourage or discourage each undergraduate to follow through with plans to go to physics graduate school. Vignettes are titled as the reason each undergraduate presented for reconsidering physics graduate school. Percentages indicate the proportion of professors who encouraged (blue) or discouraged (red) in response to each possible pairing of respondent gender and vignette. Across all vignettes, a substantially greater proportion of female professors chose to encourage relative to male professors.

To test our first, second, and third hypotheses, that the gender of the undergraduate and the professor’s gender and departmentaliation (STEM or non-STEM) impact their choice to encourage an undergraduate, we built a mixed effects logistic regression with the professor’s choice as the outcome and vignette type, undergraduate gender, and professor gender as predictors (Table 1). The ‘match’ vignette was the baseline for our model. We found that, on average professors had 47% higher odds of encouraging female than male undergraduates (OR 1.47; 95% CI: 1.003, 2.19; *p* = 0.05). Likewise, across all three vignettes, a larger proportion of professors chose to encourage female undergraduates more often than males across all three vignettes (Figure 2). These results also show that female professors were 43% less likely to encourage undergraduates than male professors (OR 0.57; 95% CI: 0.38, 0.83; *p* = 0.004). By the same token, for all vignettes, a smaller portion of female professors chose to encourage relative to male professors (Figure 3). Finally, professors affiliated with a STEM department had 300% higher odds of encouraging undergraduates (OR 3.11; 95% CI: 2.05, 4.95; *p* < 0.001).

**TABLE 1:** Logistic regression predicting whether a professor encourages an undergraduate to follow through with plans to go to physics graduate school. Professors had higher odds of encouraging if the professor was affiliated with STEM department(s), if the professor was male, when the undergraduate was a woman and when the undergraduate showed signs of “matching” with academic physics (baseline condition for the model included the one “match” vignette). The likelihood ratio test compares the target model to a null-intercept model. Number of total observations: 1,092. Number of encouraging observations: 692. Number of discouraging observations: 400. Number of respondents: 364. OR = odds ratio, P = *p*-value, 95% CI for OR = 95% confidence interval for odds ratio.

Variable	OR	*P*	95% CI for OR
Vignette: Loss of interest	0.004	<0.001	[0.001; 0.01]
Vignette: Conflicting personal goals	0.009	<0.001	[0.003; 0.02]
Gender of student: Female	1.47	0.05	[1.003; 2.19]
Professor department: STEM	3.11	<0.001	[2.05; 4.95]
Professor gender: Female	0.57	0.004	[0.38; 0.83]
**Random effects**	**SD**	—	—
Respondent ID	0.95	—	—
Overall model evaluation	${\tilde{\chi }}^{2}$	***df***	***P***
Likelihood ratio test	448.12	5	<0.001

To test our fourth hypothesis, that professors in STEM department(s) are more likely to encourage female than male undergraduates, we added to our model an interaction term between professor’s department (STEM or non-STEM) and student gender (Table 2). However, we did not find evidence that a professor’s STEM departmental affiliation increased the likelihood of encouragement for female over male undergraduates (LRT:*χ* ^2^ = 2.59, df = 1, *p* = 0.11).

**TABLE 2:** Logistic regression predicting whether a professor encourages an undergraduate to follow through with plans to go to physics graduate school, testing for whether the effect due to whether a professor was affiliated with STEM department(s) depends on undergraduate gender. We found no evidence of a conditional effect. Predictors were identical to those used for our first model (Table 1), with the added interaction term between undergraduate gender and STEM/non-STEM departmental affiliation. The likelihood ratio test compares the target model to a null-intercept model. Number of total observations: 1,092. Number of encouraging observations: 692. Number of discouraging observations: 400. Number of respondents: 364. OR = odds ratio, P = *p*-value, 95% CI for OR = 95% confidence interval for odds ratio.

Variable	OR	*P*	95% CI for OR
Vignette: Loss of interest	0.004	<0.001	[0.001; 0.01]
Vignette: Conflicting personal goals	0.009	<0.001	[0.003; 0.02]
Gender of student: Female	1.82	0.01	[1.15; 2.95]
Professor department: STEM	4.41	<0.001	[2.42; 8.53]
Professor gender: Female	0.558	0.003	[0.37; 0.82]
(Interaction) Gender of student, professor department	0.508	0.11	[0.22; 1.16]
**Random effects**	**SD**	—	—
Respondent ID	0.94	—	—
Overall model evaluation	${\tilde{\chi }}^{2}$	***df***	***P***
Likelihood ratio test	450.71	6	<0.001

## 4 Discussion

We are now decades into campaigns to create what one oft-cited national report called “an environment of encouragement” for women in STEM (Hill et al., 2010). We found evidence that, at least within academia, this project is succeeding. Our data show professors preferentially encourage undergraduate women to continue to pursue academic physics over identically described men (Figure 2; Table 1). These results echo other recent work suggesting attitudes held by STEM academics are increasingly welcoming to women–studies showing that, at least in some cases, longstanding bias in academic STEM hiring has given way to equal treatment, if not preference for women (Ceci and Williams, 2015; Williams and Ceci, 2015).

Our results suggest increasing female representation in the STEM academy is a genuine priority of professors. Reshaping the demographics of the STEM academy calls for trading-off other goals for making career choices. Our data show professors are willing to make such a trade-off. Professors appear to consider it an important goal to give career advice in advisees’ best personal interests. It is popularly believed that those careers to which we “match” are those in our best interests, and, likewise, our data show professors’ advice was strongly shaped by whether an undergraduate showed signs of “matching” or “mismatching” with academic physics (Figure 2; Table 1) (Zichy and Bidou, 2007; Christen and Bolles, 2011; Tieger et al., 2014). Our data also suggest professors strive to increase female retention in STEM at a cost to that goal. Female undergraduates had higher odds of receiving encouragement to pursue academic physics relative to men, net of vignette and other controls (Table 1).

Concretely, our results suggest that the trade-off professors are willing to make between striving to “match” and increasing female retention is this: for both undergraduates who claim they have permanently lost interest in physics and those who claim academic physics conflicts with their personal goals, professors preferentially encourage women to nevertheless pursue physics graduate school (Figure 2; Table 1). Should we be troubled that women who express such strong reasons for leaving physics are nonetheless preferentially encouraged to stay the course, compared to men who express the same reasons? Should we laud every means to “seal the leaks,” including those that make us less concerned, relative to men, to help women choose careers in their best personal interests? These questions are beyond the scope of our study, but worth consideration.

In the search for strategies to support women in STEM, research has hitherto focused on how STEM academics influence women’s decisions to continue on the path toward STEM professorship (Moss-Racusin et al., 2012; Milkman et al., 2015; Hill et al., 2010; Trower, 2008; Margolis and Miller, 2001; Whitten et al., 2007). STEM academics undoubtedly play an important role. We found no evidence, however, that non-STEM professors preferentially encourage undergraduate women to a lesser degree than do STEM professors (Table 2). In whatever ways preferential encouragement of women may be leveraged to support females in academic STEM, our work suggests non-STEM academics may be a resource. This could be valuable for diversifying the STEM academy, because many United States undergraduates who ultimately pursue STEM enter the university as undeclared or non-STEM majors. (Xie and Shauman, 2003; NAS, 2014; ACT, 2017). The non-STEM professors with whom these undeclared or non-STEM majors often interact could catalyze more women to pursue STEM.

Our study is not without limitations. First, though we sought to investigate attitudes about women pursuing STEM academia, our vignettes only depicted undergraduates in physics reconsidering plans to continue on to a physics PhD. Attitudes may vary for other STEM fields and other junctures on the career/educational path toward STEM academia. Second, though our study necessitated that we only capture some of the effect of how professors evaluate students based whether students “match” or “mismatch,” our study would benefit from a validated construct of our proposed “match” vs “mismatch” dimension (Borsboom et al., 2004; Kane, 2013). We neither know where professors perceived our vignettes to fall along our proposed dimension, nor were our vignettes designed to portray a wide array of points along this dimension. How much professors’ advice shifted due to undergraduate gender, however, could be highly sensitive to the extent to which professors perceived our students to be “match” or “mismatch” cases. Indeed, studies have shown stereotypes, including gender stereotypes, most likely shape our evaluations when other criteria do not clearly suggest what conclusion we should draw (Barrantes and Eaton, 2018; Heilman, 2012), with ambiguous cases serving as “bias amplifiers” (Tetlock and Boettger, 1989). Recent research on the effects of applicant gender on STEM hiring decisions underscores the possible importance of this on our results: While one recent study found evidence of gender bias at play for candidates with a mix of application strengths and weaknesses (Williams and Ceci, 2015), another found a clear preference for female applicants between equally unmistakably strong candidates (Ceci and Williams, 2015). Without a validated “match” vs “mismatch” construct, what our results suggest about how professors place importance on female retention relative to “matching” is quite limited.

We tested only one way a priority on “sealing the leaks” could manifest within the academic community. Of course, faculty members may be eager to encourage undergraduate women to enter STEM academia, while they and the academy remain systematically and attitudinally biased against women in STEM in other ways. We hope that the evidence we found that academics preferentially encourage women to pursue physics academia more than men will help distinguish where gender bias in STEM persists and, more broadly, help the academy fine-tune its diversity initiatives to best support women in STEM.

## Data Availability

The dataset generated and analyzed for this study, as well as the code for all analyses and figures, can be found in the Harvard Dataverse at https://doi.org/10.7910/DVN/NVCCE8.
